# Prevalence of hypertension in relation to anthropometric indices among secondary adolescents in Mbarara, Southwestern Uganda

**DOI:** 10.1186/s13052-020-00841-4

**Published:** 2020-06-01

**Authors:** Godfrey Katamba, David Collins Agaba, Richard Migisha, Agnes Namaganda, Rosemary Namayanja, Eleanor Turyakira

**Affiliations:** 1 Department of Physiology, King Ceasor University, P.O Box 88, Kampala, Uganda; 2 Department of Physiology, Mbarara University of Science and Technology, P.O Box 1410, Mbarara, Uganda; 3 Department of Community Health, Mbarara University of Science and Technology, P.O Box 1410, Mbarara, Uganda

**Keywords:** Adolescents, Hypertension, Prevalence, Anthropometric indices

## Abstract

**Background:**

Studies investigating the prevalence of hypertension and its correlation with anthropometric indices among adolescents are still scarce compared to those conducted in adults of greater than 40 years. So far, no other study estimating the prevalence and correlates of hypertension among adolescents in Uganda has been found.

**Objective:**

The purpose of this study, therefore, was to asses the prevalence of hypertension and its correlation with anthropometric indices among adolescents in Mbarara Municipality, southwestern Uganda.

**Methods:**

A cross-sectional study was carried out among 616 secondary school adolescents aged 12–19 years in Mbarara Municipality, Uganda. Blood pressure and anthropometric indices were determined by standard methods. In the statistical analysis, linear regression analysis was done to assess the relationship between blood pressure and anthropometric indices.

**Results:**

Overall prevalence of hypertension among adolescents was at 3.1% (*n* = 19) while prehypertension was 7.1% (*n* = 44). There was a statistically significant correlation between blood pressure, neck circumference, waist to hip ratio and body mass index at bivariate analysis. In multivariate analysis for anthropometric indices and sex, only neck circumference remained significantly correlated with blood pressure (*p* < 0.05).

**Conclusion:**

The prevalence of hypertension among adolescents in the study setting was low. An increase in neck circumference results in an increase in blood pressure among adolescents.

## Introduction

Hypertension (HTN) is a noncommunicable disease (NCD) which was reported to be most prevalent in Africa at 46%, followed by America at 35% [1]. Since the year 2000, an increasing upward trend of Htn has been observed [2–4] especially in low- and middle-income countries [1]. Between 60 and 100% of the disability-adjusted life-years in sub-Saharan African population is attributed to this burden [5] .

Although hypertension had been previously known as a disease for adults of 40 years or older, it is becoming common in children and adolescents [6, 7]. In the United States, an estimated 4% of adolescents between 12 and 19 years are hypertensive [8] compared to 9·5% of a similar age-group of 13 to 19 year-olds in Africa [9]. Prevalence of hypertension in adolescents is quite variable ranging from 3 to 40% [10–12] in the respective countries such United states of America, India, China, HongKong among othere. However, the majority of these studies included a wide age-range of the participants and hence can not sufficiently estimate the prevalence of Htn in adolescents aged 12-19 years. In Uganda, studies investigating the prevalence of Htn among adolescents strictly are still scarce compared to those in adults. In view of the rising burden of Htn in Africa, epidemiological studies are vital to providing evidence which is essential for planning appropriate interventions. A recent study conducted in both Uganda and Tanzania reported a prevalence of 11% among adolescents and young people [13]. The national Asthma survey which included participants aged ≥12 years, showed that the prevalence of Htn increased with age [14]. However the majority of the participants in the survey were above 19 years to reliably inform the extent of the burden in adolescents. Without the prevalence data on the silent killer among adolescents, it becomes very difficult to justify the screening for high-risk persons.

An association between adolescent hypertension and obesity was reported in several countries such as China, Korea, HongKong, Nigeria, India among others [15, 16]. Obesity is a function height and weight, but other anthropometric indices such as neck circumference waist-to-hip ratio may be important determinants of hypertension. Therefore, this study aimed at estimating the prevalence of Htn in relation to the anthropometric indices among secondary school adolescents aged 12–19 years. This was in order to clarify whether targeted screening of this age group for Htn is justifiable in peri-urban low-income settings.

## Methods

### Study setting

The study was conducted in three peri-urban secondary school schools in Mbarara Municipality in southwestern Uganda, between May and November 2018. Mbarara Municipality has up to 25 secondary schools, each with an average of 300 students. The secondary schools are both single-sex and mixed, with provisions for day schooling and boarding sections.

### Source population

This study comprised of both boys and girls aged 12–19 years, attending a secondary school within the municipality. Multistage sampling was used to select the three schools and classes in each school ranged from senior one to senior six. Volunteer sampling was used to arrive at a sample of 616 participants.

### Sampling and participant enrolment

A multistage random sampling method was used. The secondary schools in Mbarara municipality were stratified into three strata based on fees structure in the boarding section; High (≥Ugx.650, 000), Middle (Ugx.500,000 — Ugx.650,000) and lower (≤Ugx.500,000). One school was selected from each stratum by simple random sampling and from each selected school volunteer participants from Senior one to Senior six were enrolled in the study. Each of the three schools contributed about one-third of the study sample and within the school, each class contributed at least 15% of the school’s allotted number of participants. Students who are physically disabled were excluded because their height could not be easily measured.

### Sample size estimation

The sample size was estimated using the Kish Leslie method of 1965 [17], assuming a prevalence of 10.7% among secondary school adolescents [12], and 95% confidence interval within a 3% error margin. The final sample size was adjusted for an anticipated participant non-response rate of 10%; thus 449 participants were required.

### Sociodemographic information

Socio-demographic data including age, use of alcohol, individual life history of smoking and family history of hypertension were obtained through self-report.

### Anthropometric measurements

These were described in detail by Katamba et al. [18]. Height was measured using a wall mount height board without shoes in centimeters (cm) [2] with the participant shoeless and rear body parts touching the board while the head is facing forward. Weight was measured to the nearest 0.5 kg using a standard weighing scale (Seca 762, GmbH & Co. KG, Hamburg, Germany) and participants were encouraged to put on light clothing with no items in the pockets and shoeless [3]. Body mass index (BMI) was computed from the height and weight of each participant as the ratio of the weight of an individual in kilograms to height in square meters and recorded in kg/m^2^. Waist circumference (WC) was measured at the midpoint between the lowest border of the rib cage and the top of the lateral border of the iliac crest during minimal expiration the nearest 0.1 cm by an inelastic flexible measuring tape with the participant standing. Hip circumference (HC) was measured at the greatest horizontal circumference below the iliac crest at the level of the greater trochanter to the nearest 0.1 cm using an inelastic flexible measuring tape with the participant standing. Waist to hip ratio (WHR) was computed as waist circumference divided by hip circumference [4]. Neck circumference (NC) was assessed as a surrogate measure for upper body adipose tissue distribution. It was measured at the level of the laryngeal prominence using an inelastic flexible measuring tape, with the subjects in the standing position and the head held erect and eyes facing forward to the nearest 0.1 cm. The cut-offs for boys and girls were 30.75 cm and 29.75 cm respectively [19]. Any values of NC above these cutoffs indicated execss adiposity.

### Blood pressure measurement

Blood pressure was measured using a digital blood pressure machine ((Scian SP-582 Digital BP Monitor, Honsun, Jiangsu, China (Mainland) as described by Katamba et al. [18],. The participant was allowed to seat on a chair with back supported, feet on the floor, arm supported and cubital fossa at heart level after 5 min of sitting rest without talking [6, 7]. The cuff was placed at the bare upper arm, 1 inch above the bend of the participant’s elbow. It was ensured that the tubing fell over the front center of the arm so that the sensor was correctly placed. The end of the cuff was pulled so that it was evenly tight around the arm. The cuff was placed tight enough so that only two fingertips could be slipped under the top edge of the cuff. It was made sure that the skin did not pinch when the cuff inflated. The participant was asked to remain calm and quiet as the machine begins measuring. The cuff inflated automatically after placing the start button, and then slowly deflated so that the machine took the measurement. When the reading was complete, the monitor displayed the BP on the digital panel [8]. Three readings were recorded per participant at 5 min’ interval. The average of the 2nd and 3rd SBP and DBP measurement were used as the subject’s BP respectively [7]. Those adolescents who had elevated BP in the first session were identified. A re-measurement, using the same procedure was done after 1 week to confirm that BP is truly and constantly elevated.

### Blood pressure classification

Hypertension was defined as BP value ≥95th percentile of the BP for the gender, age, and height for adolescents less than 18 years while preHTN were defined as SBP/DBP ≥ 90th percentile but less than 95th percentile according to recommendations from the European society of HTN and Fourth report on management of HTN [20, 21]. Adolescents aged 18–19 years were considered as adults and HTN among them was classified as average SBP ≥ 130 mmHg and average DBP ≥ 80 mmHg. Prehypertension was defined as SBP of 120–129 mmHg and DBP < 80 mmHg respectively, according to the 2017 American College of cardiology and American Heart association guidelines on hypertension [22].

### Data management and analysis

All data were recorded on paper forms, entered into Excel (Microsoft office 2013) and analyzed using Stata software version 13.0 (College Station, Texas, USA). Association of Htn with behavioral factors was analyzed using the logistic regression analysis, reporting their odds ratios (OR) and respective 95% confidence interval (CI). The correlations of blood pressure with anthropometric indices were determined using linear regression analysis; beta coefficients and respective confidence intervals are reported. A *p*-value < 0.05 was considered statistically significant.

## Results

### Characteristics of study participants

A total of 616 secondary school adolescents participated in this study, including 212 boys and 404 girls. They were aged between 12 to 19 years with a mean age of 15.6 years and an average BMI of 23.9kgm^− 2^. Females were slightly heavier than boys. The mean systolic blood pressure (SBP) was slightly higher in girls as compared to their counterparts as showed in Table 1.

**Table 1:** Characteristics of the study participants by sex

Variable	Girls (*n* = 404)	Boys (*n* = 212)	Total (*n* = 616)
	*Mean (SD)*	*Mean (SD)*	*Mean (SD)*
Age (*years*)	15.8 (1.9)	15.3 (2.1)	15.6 (2.0)
Weight (*kg*)	61.2 (8.2)	56.3 (8.0)	59.5 (8.4)
Height (*cm*)	157.5 (7.0)	158.7 (8.3)	157.9 (7.5)
Hip Circumference (*cm*)	94.5 (10.7)	81.5 (9.2)	90.0 (11.9)
Waist Circumference (*cm*)	71.7 (7.9)	68.4 (6.7)	70.8 (8.0)
Body Mass Index (*kgm*^*− 2*^)	24.7 (2.7)	22.4 (3.0)	23.9 (3.0)
Neck Circumference (*cm*)	30.0 (1.6)	30.2 (2.8)	30.0 (2.1)
Waist to Hip Ratio	0.76 (0.07)	0.84 (0.06)	0.79 (0.08)
Waist to Height Ratio	0.46 (0.05)	0.43 (0.04)	0.44 (0.05)
Resting Pulse Rate (*bpm*)	76.5 (8.4)	73.8 (8.0)	75.5 (8.3)
Systolic Blood Pressure (*mmHg*)	114.0 (7.3)	112.0 (8.0)	113.3 (9.0)
Diastolic Blood Pressure (*mmHg*)	66.8 (8.2)	65.9 (8.0)	66.5 (8.1)

*SD* standard deviation

### Prevalence of hypertension among adolescents

The overall prevalence of hypertension among adolescents was determined as 3.1% (*n* = 19) while prehypertension was 7.1% (*n* = 44). Prevalence of prehypertension was lower in girls at 6.4%(*n* = 26) compared to 8.5% (*n* = 18) among the boys. The overall prevalence of systolic hypertension was 5.2% (*n* = 32) while that of diastolic hypertension was at 9.4% (*n* = 58). The values in Table 2 shows the proportion of systolic and diastolic hypertension by sex of the participants.

**Table 2:** Prevalence of hypertension by sex

BP	Boys; n (%)	Girls; n (%)	Total; n (%)
Systolic			
Normal	197 (92.9)	387 (95.8)	584 (94.8)
Htn	15 (7.1)	17 (4.2)	32 (5.2)
Diastolic			
Normal	193 (91.0)	365 (90.3)	558 (90.6)
Htn	19 (9.0)	39 (9.7)	58 (9.4)

*Htn* Hypertension

### Correlates of hypertension among adolescents

Having a personal history of taking alcohol and a positive family history of hypertension were significantly associated with hypertension at both bivariate and multivariate analyses. The details of behavioral factors for hypertension are summarised in Table 3.

**Table 3:** Association of behavioural factors with hypertension

History	Hypertension		OR [CI]	p-value	aOR [CI]	p-value
	Yes *n (%)*	No *n (%)*				
Ever taken alcohol						
*Yes*	4 (12.5)	28 (87.5)	5.4 [1.7–17.4]	0.005*	5.1 [1.5–17.1]	0.009*
*No*	15 (2.6)	569 (97.4)				
Family History of hypertension						
*Yes*	7 (8.9)	72 (91.1)	4.3 [1.6–11.1]	0.003*	4.1 [1.5–11.0]	0.005*
*No*	12 (2.2)	525 (97.8)				
Ever smoked						
*Yes*	0	11 (100.0)	Omitted		Omitted	
*No*	19 (3.0)	586 (97.0)				

*OR* Odds ratio, *CI* Confidence interval, *aOR* adjusted odds ratio, *;significant

Analysis of the possible linear relationship between blood pressure measurements and anthropometric indices revealed that both systolic and diastolic blood pressure significantly increased with increase in neck circumference among adolescents. Although a unit increase in body mass index was associated with a significant increase in both systolic and diastolic blood pressure at bivariate analysis, the effects (coefficients) became smaller and non-significant after adjusting for other anthropometric indices. Unexpectedly, a unit increase in waist to hip ratio resulted in a decrease in diastolic blood pressure though it was not statistically significant at multivariate analysis (Table 4).

**Table 4:** Linear regression of anthropometric indices with blood pressure

Variable	Bivariate		Multivariate (adjusted; NC,WHR,BMI, sex)	
	Beta [CI]	*p*-value	aBeta [CI]	*p*-value
SBP				
BMI (kgm^−2^)	0.31 [0.07;0.54]	0.011*	−0.15[−0.38;0.07]	0.184
WHR	−16.3[−25.7;-7.0]	0.001*	−3.65[−13.1;5.83]	0.450
NC (cm)	0.61 [0.54;0.68]	< 0.001*	2.23 [1.93;2.54]	< 0.001*
DBP				
BMI (kgm^−2^)	0.12[−0.09;0.33]	0.265	−0.10[− 0.32;0.12]	0.373
WHR	−15.4[−23.8; −7.0]	< 0.001*	−11.7[− 21.1;-2.32]	0.015*
NC (cm)	1.25 [0.96;1.54]	< 0.001*	1.25 [0.95;1.56]	< 0.001*

*BMI* Body mass index, *NC* Neck circumference, *WHR* Waist hip ratio, *SBP* Systolic blood pressure, *DBP* Diastolic blood pressure, *CI* Confidence interval, *aBeta* adjusted beta coefficient; *; significant

## Discussion

The current study found that the prevalence of hypertension among secondary school adolescents in the study setting was low. However, the prevalence was more in boys as compared to girls. High prevalences of adolescent hypertension have been reported in populations where the prevalence of obesity was also high [10, 23]. However, in our study setting, the prevalence of obesity was equally low with only 3.4% of study participants classified as obese.

In our study setting most participants had regular exercise as they walked to and from school, or within their school environment. Studies have shown variations in the prevalence of hypertension by geographical region, level of physical activity as well as socio-economic status. Some adolescents even in a school setting do not participate in co-curricular activities due to a tight academic schedule; they spend much of their time seated and studying week in week out [24]. This denies them the opportunity to do minimal exercises to improve cardiovascular fitness. Others while on holiday, feed on an unhealthy diet which is high in fat content, inadequately feed on fruits and vegetables [25] in addition to watching more television [26, 27] and hence becoming overweight and obese. The diet consumed by the adolescents especially salty food and modified foods like the use of energy drinks [28, 29] and consuming alcohol are also likely causes as was evidenced that some adolescents reported ever use of alcohol. However, the details of how much alcohol drunk and salt consumed were not investigated in this study. Other lifestyle factors such as exposure to persistent academic stress have been identified among adolescents and indicated as risk factors for hypertension [30]. The other probable cause of high blood pressure in adolescents could be persistent hyperactive sympathetic nervous system even at rest [31–33], which increases the smooth muscle tone of the vessels increasing resistance and hence persistently elevating pulse rate, cardiac output and hence elevating blood pressure [34, 35]. In the current study participants were not screened for any underlying chronic infections like endocrinopathies which have been linked to high blood pressure [36, 37].

Our study findings differ significantly from the findings by several studies which reported prevalences ranging from 3 to 40%. In India, a study reported a prevalence of adolescent hypertension of 21.5% [38], which was almost 8 times higher than in the current study. In Nigeria, Uwaezuoke and colleagues reported a prevalence of adolescent hypertension of 10.7% [12] which was almost 4 times that of the current study. This differences may be due to overdiagnosis of blood pressure in the Nigeria study which arising from short-time-interval between measurements and taking all measurements on the same day. In our study, participants with elevated blood pressure on the first contact were re-assessed after 1 week to ensure the accuracy and reliability of the recorded values. Other findings in Uganda from a study which involved participants of 15 years and older, found a prevalence of 27.4% [11]. However, in this study, 70 % of the participants were aged more than 24 years and so the prevalence estimated in this study cannot generalized to all adolescents in Uganda. Findings from the current study are consistent with other studies of adolescents of adolescents in Houston schools in the USA where McNiece and colleagues reported a prevalence of 3.2% after three screenings [39] w. Nsanya and colleagues reported a prevalence of 11% from a study which included adolescents and young adults 12 to 24 years old from Uganda and Tanzania. The difference in the participant’s age would have created the difference between the studies. The national Asthma study also reported the prevalence of Htn increased with age in a study which involved all participants aged ≥12 years.

Whereas several studies on Htn among adolescents have singled out childhood obesity as a key driver of adolescent hypertension [6, 40–42], we were unable to demonstrate a significant relationship between BMI and blood pressure in our study. However, we found that neck circumference was a more important predictor of change in blood pressure in the currentr study. In Greece, study findings indicated that all anthropometric indices (AIs) measured including; body mass index (BMI), neck circumference (NC), waist circumference (WC), hip circumference (HC) waist-hip ratio (WHR) and waist to height ratio (WHtR) were correlated with SBP and DBP. Neck circumference was reported to be associated with most CVD risk factors [43]. However, only children of 9 to 13 years were studied and the sample size was too small to draw reliable conclusions. A study in India reported that Htn was associated with a greater mean weight, BMI and waist circumference [44]. In this study, other AIs were not assessed and the sample size was small to make definitive conclusions. A study among Korean adolescents found that high BMI (>85th percentile) and high waist circumference (>90th percentile) were positively correlated with high SBP (>90th percentile) in both sexes and high DBP (>90th percentile) in boys [45]. A community-based cross-sectional study reported that an increase in SBP and DBP was correlated with an increase in neck circumference and BMI [46]. From the United States of America, findings showed that odds ratios for elevated BPs were higher in children with wide neck circumference than those with normal neck circumference. BMI and neck circumference were associated with elevated BP in children of 8 to 18 years [47]. Our study was limited by funding and hence could not study the role of endocrinopathies and dyslipidemia in adolescent hypertension. Additionally, data on the correlation between heart rate and blood pressure was not analysed.

## Conclusions

Hypertension was less common among adolescents in this study. Blood pressure was positively correlated with anthropometric indices except for waist-height ratio.

## Data Availability

The dataset is available on request from the corresponding author.

## Abbreviations

BP: Blood pressure
NCD: Non communicable disease
BMI: Body mass index
WC: Waist circumference
HC: Hip circumference
NC: Neck circumferebce
SBP: Systolic blood pressure
DBP: Diastolic blood pressure
CI: Confidence interval
CVD: Cardiovascular disease
WHR: Waist hip ratio
OR: Odds ratio
aOR: Adjusted odds ratio
